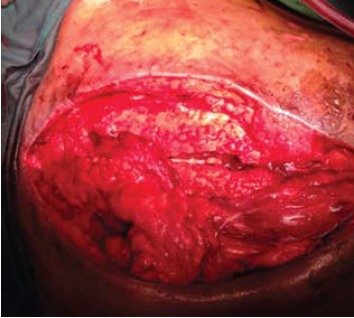# Trapezius Rotational Flap for Cervico-thoracic Wound Breakdown in Post-radiotherapy Necrosis : A Case Report

**DOI:** 10.5704/MOJ.1407.001

**Published:** 2014-07

**Authors:** MH Ariffin, Selvyn Lloyd, SA Rhani, Kamalnizat, A Baharudin

**Affiliations:** Department of Orthopaedic and Traumatology, University Kebangsaan Malaysia Medical Centre, Kuala Lumpur, Malaysia; Department of Orthopaedic and Traumatology, University Kebangsaan Malaysia Medical Centre, Kuala Lumpur, Malaysia; Department of Orthopaedic and Traumatology, University Kebangsaan Malaysia Medical Centre, Kuala Lumpur, Malaysia; Department of Orthopaedic and Traumatology, University Kebangsaan Malaysia Medical Centre, Kuala Lumpur, Malaysia; Department of Orthopaedic and Traumatology, University Kebangsaan Malaysia Medical Centre, Kuala Lumpur, Malaysia

## Abstract

The management of post-radiation wound breakdown over the posterior cervico thoracic region can be a challenging task for a surgeon. The aim of the treatment is to produce a well vascularized and a low tensile flap which will close a large defect. We describe the use of the lower trapezius flap to reconstruct the wound breakdown and to obtain stable tissue coverage in a patient with postradiation necrosis. This flap minimizes the disruption of the scapula-thoracic function while preserving the range of movement over the shoulder. From the literature review, it was noted that the dorsal scapular artery (DSA) and transverse cervical artery (TCA) aid in the blood supply to the trapezius muscle and prevent local necrosis during rotation of the flap. The trapezius flap is widely accepted because of the minor donor site morbidity, large arc of rotation and adequate blood supply.

**Key Words:**

lower trapezius flap, scapula-thoracic function, postradiation necrosis

## Introduction

Post-radiation wound break down over the posterior aspect of the cervico-thoracic region can lead to significant morbidity and disfigurement. The trapezius and the latissimus dorsi muscles components can be used as flap transfer to address the large defect. The use of latissimus dorsi flap alters the glenohumeral function and this leads to reduction in the range adduction and extension over the shoulder, causing shoulder fatigue.^1^ Hence, a better understanding of the vascular anatomy of the trapezius muscle led to the development of the musculocutaneous trapezius flap.

## Case Report and Surgical Procedure

**Fig. 1: Wound breakdown with exposed implant over the cervico- thoracic area.**
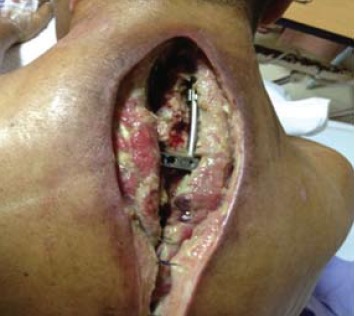


A 52 year old man was diagnosed with adenocarcinoma of the lung a year previously. He presented with bilateral upper limb pain and weakness for six months. Magnetic resonance imaging (MRI) revealed multiple enhancing lesions with pedicle involvement and collapse of Cervical 5 (C5) vertebra with spinal cord compression and also metastasis to Thoracic 7 vertebral body without any collapse or cord compression at that level. Patient was then planned for elective resection of the C5 vertebral body lesion and lateral mass fixation from C3 to C7. One month after the index surgery, patient was readmitted because of back pain and progressive weakness over the lower limbs. The radiological imaging on the second admission showed a pathological fracture at Thoracic 7 (T7) vertebra. Subsequently, T7 vertebrectomy, and vertebral body replacement with posterior stabilization was done via a posterior costotranverse approach. On the 10th post-operative day, the patient was started on adjuvant radiotherapy. A single fraction radiotherapy was given on 20th post operative day . A week later patient presented with wound breakdown at the cervico-thoracic junction. Wound debridement was done for the cervico-thoracic region and the implant was exposed. All tissue and swab culture and sensitivity tests during initial debridement were negative. Attempts at using vacuum assisted closure failed due to the massive size of the defect and the exposed implants (Figure 1). Patient was counselled for trapezius rotational flap for which he consented.

**Fig. 2: After 1st wound debridement over cervico- thoracic region with exposed implant.**
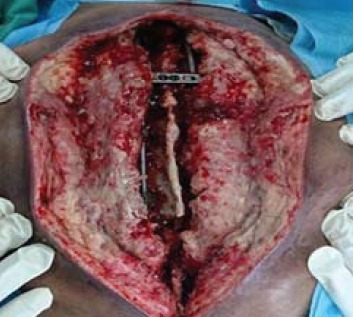


**Fig. 3: Shows the lower trapezius dissected from the spinous process of T12 and rotated superiorly to close the defect.**
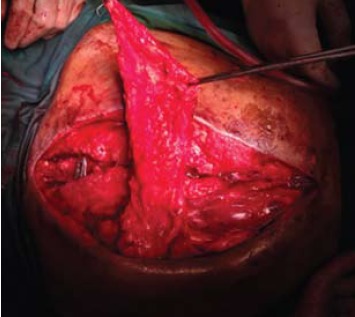


Under general anaesthesia, the patient was placed in prone position over the Jackson table. The wound over the back was cleaned and was extended distally followed by subcutaneous dissection to expose the inferior half of the muscle on the left side (Figure 2). The left trapezius insertion site was identified over the distal aspect of the T12 spinous process and the trapezius muscle was exposed proximally till the inferior angle of scapula by dissecting towards the scapula spine. It was found to be atrophied over the insertion site. The muscle bulk of the trapezius over the inferior angle spine appeared normal. The inferior half of the trapezius was detached from its insertion and was rotated anti clockwise superiorly to close the defect over the exposed implant (Figure 3a) and (Figure 3b). The muscle was then sutured loosely, subcutaneous tissue approximated with vicryl 1/0 and dressing applied.

The post operative period was uneventful. No necrosis of the flap occurred. The implant was well covered. The patient was discharged on the 10th post-operative day . No further radiotherapy was given as the original oncology plan was to administer a single dose radiotherapy.

## Discussion

Tissue coverage over the posterior cervicothoracic defect can be a challenging task for a surgeon. The selection of flap depends on factors such as reliable blood supply, and extension and location of the defect. The trapezius flap has been used in several procedures such as in craniofacial defect, neck defect, reconstruction of the hypopharynx and cervical oesophagus. 

The trapezius flap consists of the triangular muscle which can be divided into superior and inferior segments. The superior segment is the most important part of the muscle since it receives the spinal accessory nerve for motor innervation. The inferior part of the trapezius is known as a dispensable unit. The blood supply enters through the deep surface from the posterior descending branch of the transverse cervical artery which in turn arises from the subclavian artery. This artery can be rotated without compromising the blood supply to fill the defect over the cervicothoracic region.

Since 1980, the lower pedicular flap has been used as a standard muscular cutaneous flap in neck and head reconstruction. According to the classification of Mathes and Nahai, the trapezius has Type 2 vascular pattern with dominant and additional minor vascular pedicle ^2^ . The first description of the lower trapezius flap identified the transverse cervical artery with it superior branch as a major feeding artery. In 1991, Netterville and Wood reinvestigated the flap by dissection of 15 cadavers and found that the dorsal scapular artery was the dominant vessel in 50% and the transverse cervical artery in 30 % ^3^. Lynch et al found that the dominant vessel was the deep branch of the transverse cervical artery which was superior to the levator scapulae and rhomboid muscles 4. This formed a standardize nomenclature of these vessels which would help to achieve a safe flap elevation during rotation without compromising the blood supply to the muscle.

In our patient, radiation therapy over the cervicothoracic region could affect the descending branch of the transverse cervical artery located in between the medial edge of the scapula and the spine. In an anatomical study, Tan, et al have outlined the course of the dorsal scapular artery and the presence of musculocutaneous perforators of the dorsal scapular artery over the lateral edge of the trapezius muscle ^5^. This ensures adequate blood supply which facilitates closure of the large defect over the cervicothoracic region and wound healing. The trapezius musculocutaneous flap has also been used in the case of severe shoulder and neck burns with cicatrical contracture deformity. This is a relatively good method of soft tissues repair over the neck with adequate blood supply avoiding local flap necrosis after relocation.

The trapezius muscle compared with other muscles for the coverage of the posterior defect such as latissimus dorsi, has a low donor site morbidity and allows early rehabilitation. Glenohumeral function is preserved in lower trapezius flap in contrast with latissimus dorsi which result in weakness of the shoulder extension and adduction.

**Fig. 4: Shows the lower aspect of the trapezius loosely suture over the subcutaneous to cover the implant.**